# Research on the Optimization of the Electrode Structure and Signal Processing Method of the Field Mill Type Electric Field Sensor

**DOI:** 10.3390/s25134186

**Published:** 2025-07-04

**Authors:** Wei Zhao, Zhizhong Li, Haitao Zhang

**Affiliations:** 1 School of Intelligent Manufacturing, Huzhou College, Huzhou 313000, China; zhaowei@zjhzu.edu.cn; 2 State Key Laboratory for Disaster Prevention & Mitigation of Explosion & Impact, Army Engineering University of PLA, Nanjing 210007, China

**Keywords:** electric field sensor, finite element, pre-integral transformation circuit, fast Fourier transform, BP neural network

## Abstract

**Highlights:**

**What are the main findings?**

- The law of the influence of the thickness of the sensor’s buckling electrode and the distance between the sensing electrode and the buckling electrode on the sensor’s sensitivity;
- The improved FFT-BP method for harmonic noise reduction.

**What is the implication of the main finding?**

- The electrode parameters of the field mill type electric field sensor have been optimized;
- The completed mathematical model of the input and output of the field mill type electric field sensor was established;
- A processing method for the output signal of the field mill type electric field sensor was studied, and the signal processing circuit was designed and optimized.

**Abstract:**

Aiming at the issues that the field mill type electric field sensor lacks an accurate and complete mathematical model, and its signal is weak and contains a large amount of harmonic noise, on the basis of establishing the mathematical model of the sensor’s induction electrode, the finite element method was adopted to analyze the influence laws of parameters such as the thickness of the shielding electrode and the distance between the induction electrode and the shielding electrode on the sensor sensitivity. On this basis, the above parameters were optimized. A signal processing circuit incorporating a pre-integral transformation circuit, a differential amplification circuit, and a bias circuit was investigated, and a completed mathematical model of the input and output of the field mill type electric field sensor was established. An improved harmonic detection method combining fast Fourier transform and back propagation neural network (FFT-BP) was proposed, the learning rate, momentum factor, and excitation function jointly participated in the adjustment of the network, and the iterative search range of the algorithm was limited by the threshold interval, further improving the accuracy and rapidity of the sensor measurement. Experimental results indicate that within the simulated electric field intensity range of 0–20 kV/m in the laboratory, the measurement resolution of this system can reach 18.7 V/m, and the measurement linearity is more than 99%. The designed system is capable of measuring the atmospheric electric field intensity in real time, providing necessary data support for lightning monitoring and early warning.

## 1. Introduction

The atmospheric electric field intensity is an important parameter for characterizing thunderstorm activities [1]. The accuracy of atmospheric electric field intensity detection is of great significance for lightning early warning [2]. The field mill type electric field sensor has advantages such as a simple structure, accurate measurement, and low manufacturing cost, and it is widely used in the measurement of the atmospheric electric field. A miniature field meter, which was designed by Harrison, is described for measuring fair-weather electric fields [3]. Reference [4] introduced a sensor that can forecast lightning incidents, and by using this device, an electric field threshold for lightning strikes on aircraft can be defined. Zhang and Liu studied the calibration of the field mill type electric field sensor in a space charge environment [5,6]. Cui established a 3D model of the electric field sensor calibrated device using the finite element method and reduced the measurement error by a correction algorithm [7,8]. Zheng conducted a simulated analysis of the sensitive structure on the sensor and calculated the change in charge on the electrode [9]. The above studies optimized the parameters of the sensor electrode using the finite element method, laying a solid foundation for the detection of electric field intensity. However, the above studies ignored the analysis of the change law of induced charges from the analytical model, and failed to establish a complete mathematical model regarding the electric field intensity and the output voltage of the sensor.

The current signal generated by the field mill type electric field sensor is not only weak but also comprises DC components, harmonic components, and interference noise, resulting in insufficient measurement accuracy of the electric field intensity. Therefore, it is necessary to study a signal processing method in view of the characteristics of the output signal generated by the induction electrode. Agorastou introduced and analyzed the design method of the field grinding sensor interface, and discussed the related parameters such as the sensor’s sensitivity [10]. Dou designed a universal high-precision weak signal amplifier based on AD620 [11]. Cheng designed a preamplifier circuit to address the noise and temperature reliability issues in weak signal detection [12]. Chang proposed a weak signal temperature compensated method, a high-precision differential bridge circuit was adopted, and the measurement accuracy was improved [13]. However, the above studies only focused on signal amplification; the signal-to-noise ratio after signal amplification was ignored. Moreover, there are few studies on the processing methods of the output signal generated by field mill type electric field sensors.

For the problem that the signal contains a large amount of harmonic interference, the harmonic detection methods in the field of power grids can be referred to. Currently, the fast Fourier transform (FFT) [14], wavelet analysis [15], and Hilbert–Huang Transform (HHT) [16] were widely used the power harmonic detected algorithm. Wavelet analysis can lead to wavelet aliasing, causing insufficient system stability. The HHT method is prone to generating false component signals. The FFT can cause spectral leakage and the picket fence effect during asynchronous sampling and periodic truncation. All-phase FFT (ap-FFT) analysis can effectively suppress spectral leakage and prevent white noise interference, which is widely used in harmonic noise reduction [17]. Su adopted an ap-FFT algorithm with high-order window functions, and the detection accuracy of harmonics was improved [18]. But due to the requirement of two FFT transformations, the amount of computation is large. The modified ap-FFT algorithm was used, and the modified formula was simple [19,20], but when there are insufficient sampling points, the accuracy of the test results cannot be guaranteed. A harmonic detection method based on ap-FFT and BP neural network was proposed [21,22], which has certain advantages in the detection of integer harmonic signals, but because the inaccurate setting of parameters such as learning rate and momentum factor in the algorithm, the operation takes a long time and the real-time measurement of electric field intensity cannot be realized.

Therefore, in view of the problems such as the lack of an accurate mathematical model of the sensor, weak output signal, and the inclusion of a large amount of harmonic noise, based on the establishment of the electrode induction electrode analytical model, the finite element method is adopted to optimize parameters such as the thickness of the bucking electrode and the distance between the bucking electrode and the induction electrode. A signal processing circuit including an I–V integral transformed circuit, a differential amplification circuit, and a bias circuit was studied, and the amplification and conversion of the output signal generated by the induction electrode was produced. An improved filtering method combining fast Fourier transform and back propagation neural network (FFT-BP) was proposed, which effectively suppressed harmonic interference, and the rapid measurement of the electric field intensity was achieved. The electric field intensity is detected in real time under thunderstorm weather, and the measured electric field intensity is consistent with the current weather changes. The device studied in this paper can provide the necessary data support for lightning early warning.

## 2. Structural Parameters Optimized by Field Mill Type Electric Field Sensors

### 2.1. The Structure and Measurement Principle of the Sensor

**Figure 1:**
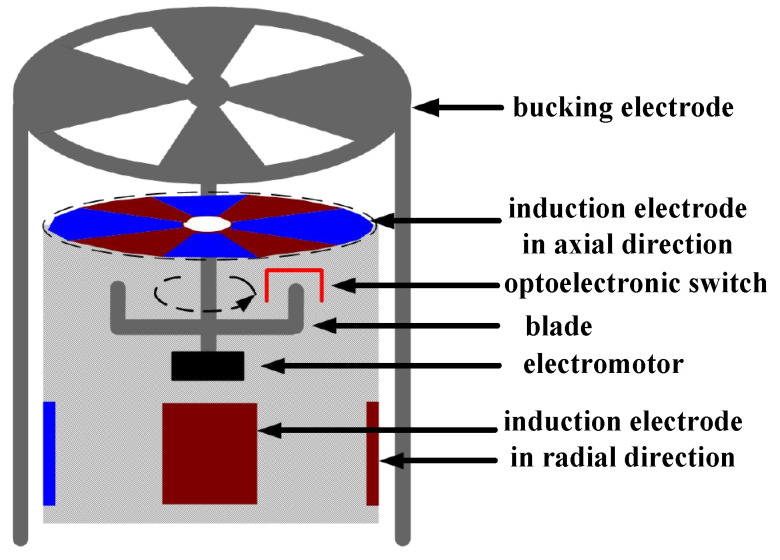
Structure of a field mill type 3D field sensor.

The field mill type electric field sensor is composed of a motor shaft, an insulating shell, three sets of induction electrodes in orthogonal directions, and a bucking electrode. The structure is shown in Figure 1, where the electrode perpendicular to the motor shaft is the axial electrode, and the other two are radial electrodes.

Measurement principle: The bucking electrode rotates at period τ that is driven by the motor shaft, and the three orthogonal direction induction electrodes are periodically exposed to the electric field environment, and the surface produces a periodic change of induced charge. Under the action of the external electric field, the induced charge on the induction electrode produces a current signal with a certain proportion of the external electric field due to its periodic change. Since this device is mainly used for the detection of ground atmospheric electric field, the ground atmospheric electric field is in the plumb direction, which is mainly measured by the axial induction electrode. Therefore, the structural optimization of the axial induction electrode under the action of the electric field in the plumb direction ${\vec{E}}_{Z}$ was studied. The induced charge of the induced electrode in the plumb direction $Q_{Z}$ can be expressed as Equation (1)

(1) $$Q_{Z}(t)\;=\;\varepsilon_{0}{\vec{E}}_{Z}A(t)$$

In Equation (1), $\varepsilon_{0}$ is permittivity of vacuum, $\varepsilon_{0}\;=\;8.85\;\times \;10^{-12}\;F/m$, $A_{Z}(t)$ is the area of the induction electrode in the Z direction, ${\vec{E}}_{Z}$ is the component of $\vec{E}$ in the direction of Z. The current signal output by the induction electrode in the Z direction $I_{Z}$ can be expressed in Equation (2)

(2) $$I_{Z}\;=\;\frac{dQ_{Z}(t)}{dt}\;=\;\varepsilon_{0}E_{Z}\frac{dA_{Z}(t)}{dt}$$

In Equation (2), $I_{Z}$ is related to $A_{Z}(t)$ and its rate of change. A fan-shaped shape is adopted for the induction electrode of the sensor, and the change rate of the electrode’s exposed area to the electric field is expressed in Equation (3).

(3) $$\frac{dA_{Z}(t)}{dt}\;=\;\left\{\begin{array}{c} \frac{\pi (r_{2}^{2}\;-\;r_{1}^{2})/2}{\tau /8}\;=\;4\pi f_{0}(r_{2}^{2}\;-\;r_{1}^{2}),\;0\;<\;t\;\leq \;T\\ -\frac{\pi (r_{2}^{2}\;-\;r_{1}^{2})/2}{\tau /8}\;=\;-4\pi f_{0}(r_{2}^{2}\;-\;r_{1}^{2}),\;T\;<\;t\;\leq \;2T \end{array}\right.$$

In Equation (3), $r_{1}$ and $r_{2}$ are the inner and outer radii of the induction electrode, τ is the motor rotation period, $f_{0}$ is the electrode rotation frequency, T is the time required for the induction electrode to be completely shielded or exposed once. The electrode edge effect is ignored, and $I_{Z}$ is expressed by Equation (4)

(4) $$I_{Z}(t)\;=\;\left\{\begin{array}{c} \frac{dQ_{Z}(t)}{dt}\;=\;-4\pi \varepsilon E_{Z}f_{0}(r_{2}^{2}\;-\;r_{1}^{2}),\;0\;<\;t\;<\;T\\ \frac{dQ_{Z}(t)}{dt}\;=\;4\pi \varepsilon E_{Z}f_{0}(r_{2}^{2}\;-\;r_{1}^{2}),\;T\;<\;t\;<\;2T \end{array}\right.$$

**Figure 2:**
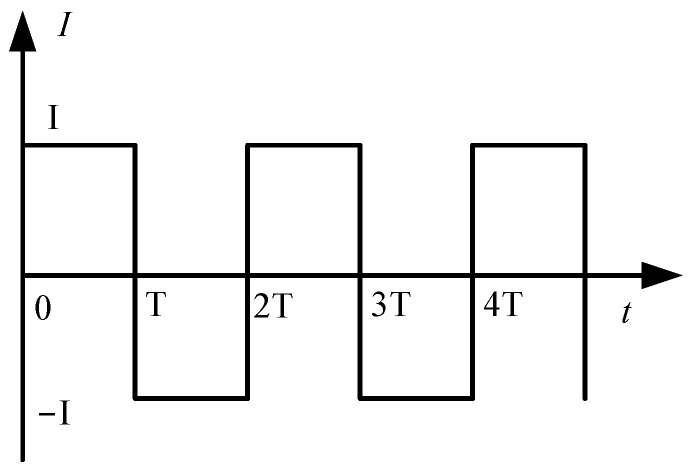
Induced current signal waveform.

By analyzing Equations (2)–(4), the induced current $I_{Z}$ output by the sensor is related to parameters such as the field intensity E, frequency of electrode rotation $f_{0}$, electrode size $r_{1}$ and $r_{2}$, etc. The larger the area of the induction electrode is, the greater the amplitude of the generated signal will be. Considering that the volume of the sensor cannot be too large, the inner and outer radii of the fan-shaped induction electrode are selected, respectively, $r_{1}\;=\;7\;\mathrm{mm}$ and $r_{2}\;=\;16\;\mathrm{mm}$. When the size of the induction electrode, the rotated frequency, and the electric field intensity are stable, the output current signal of the Z-direction induction electrode is a square wave, as shown in Figure 2.

### 2.2. The Influence of the Thickness of the Sensor Bucking Electrode, the Distance Between the Induction Electrode, and the Bucking Electrode on the Sensitivity

**Figure 3:**
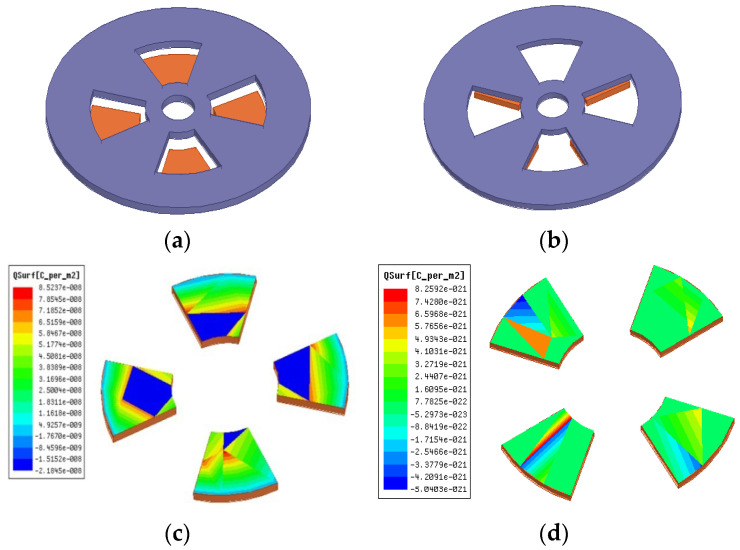
The surface charge distribution of the inductive electrode: (**a**) the induction electrode is fully exposed; (**b**) the induction electrode is completely shielded; (**c**) $Q_{1}$; (**d**) $Q_{2}$.

Due to the edge effect of the electrode, the distance between the induction electrode and the bucking electrode d, as well as the thickness of the bucking electrode h, which will directly affect the charge quantity of the induction electrode. In order to improve the sensitivity, for the measurement of the Z-direction electric field intensity, the influence of d and h on the sensor sensitivity is analyzed. Figure 3 shows the charge distribution of the Z-direction induction electrode in two states: fully exposed and fully shielded in the electric field.

**Figure 4:**
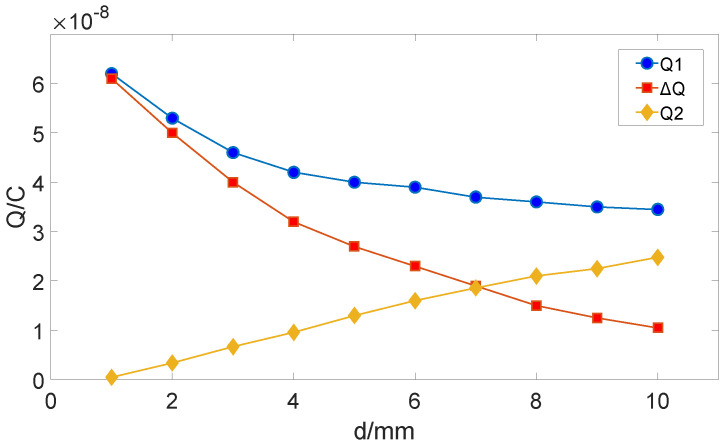
The relationship between ∆Q and d

In Figure 3, when the induction electrode is fully exposed, the induced charge quantity is $Q_{1}$; when it is fully shielded, the induced charge quantity is $Q_{2}$; the maximum change in the induced charge quantity is $∆Q\;=\;Q_{1}\;-\;Q_{2}$. The relationship between ∆Q and d is shown in Figure 4.

As Figure 4 shows, $Q_{1}$ decreases approximately exponentially with the increase of d, $Q_{2}$ increases linearly with the increase of d, and ∆Q decreases with the increase of d, showing an approximately inversely proportional relationship. Therefore, the electrode spacing of the sensor should be reduced as much as possible. Considering the installation cost of the electrodes, if d is too small, the installation difficulty will increase, so it is selected that d = 2mm.

**Figure 5:**
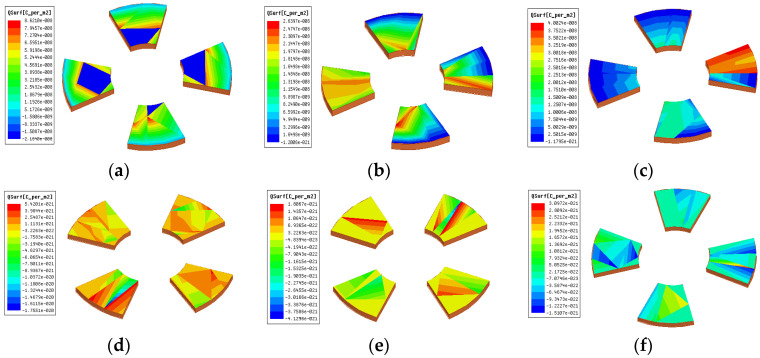
The surface charge distribution of the inductive electrode: (**a**) $Q_{1}h=1\;\mathrm{mm}$; (**b**) $Q_{1}$h=1.5 mm; (**c**) $Q_{1}$h=2 mm; (**d**) $Q_{2}$h=1 mm; (**e**) $Q_{2}$h=1.5 mm; (**f**) $Q_{2}$h=2 mm.

When d = 2 mm, $r_{1}\;=\;7\;\mathrm{mm}$, $r_{2}\;=\;16\;\mathrm{mm}$, the thickness of the bucking electrode is selected as 1 mm, 1.5 mm and 2 mm, respectively, the influence on the surface charge change of the induction electrode was studied. The charge distribution on the surface of the induction electrode is shown in Figure 5.

**Figure 6:**
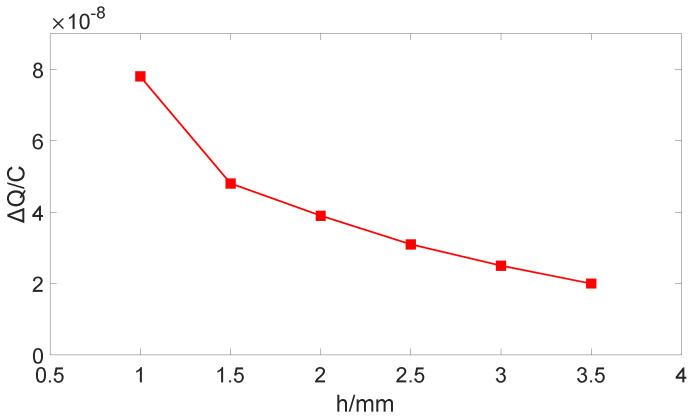
The relationship between ∆Q and *h*.

The relationship between ∆Q and h is shown in Figure 6.

As shown in Figure 5 and Figure 6, the greater the thickness of the bucking electrode is, the less change in the induced charge on the electrode is. Therefore, the thinner bucking electrodes are selected to enhance the sensitivity of the sensor. Comprehensively considering the fabrication and installation of the sensor induction electrode and the rules obtained from the above simulation, the final determined parameters are: d = 2 mm, h = 1 mm, $r_{1}\;=\;6\;\mathrm{mm}$, $r_{2}\;=\;17\;\mathrm{mm}$.

## 3. Signal Processing Method for Field Mill Type Electric Field Sensors

**Figure 7:**
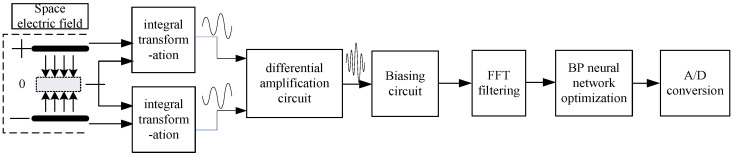
Signal processing flow.

Since the signal generated by the induction electrode is very weak and contains interference noises such as DC components and a large number of harmonic components, amplitude measurement can only be carried out after amplification, filtering, and A/D conversion. The processing flow of the sensor output signal is shown in Figure 7. Firstly, the two induced current signals generated by the induction electrode are subjected to I–V transformation, converting the weak current signal into a voltage signal and amplifying it. Secondly, the voltage signal is successively passed through the differential amplification circuit and the bias circuit to reach the effective range of A/D conversion. Finally, the improved FFT-BP method is adopted to decompose the signal according to different frequencies to reduce the harmonic interference. After decomposing the effective voltage signal, A/D conversion and amplitude measurement are carried out, and the measured electric field intensity is obtained by the established analytical model of electric field intensity and voltage amplitude.

### 3.1. Pre-Integral Transformation Circuit

#### 3.1.1. Analytical Model of Pre-Integral Transformation Circuit

The current I generated by the induction electrode is taken as the input of the pre-integral transformation circuit, and the voltage U after transformation is the output of the circuit. The analytical model of the circuit is shown in Equation (5)

(5) $$U(t)\;=\;I\cdot R\;-\;RC\frac{dU(t)}{dt}$$

In Equation (5), R is the equivalent resistance of the circuit, C is the equivalent capacitance, and U(t) is obtained as Equation (6).

(6) $$U(t)\;=\;I\cdot R\;-\;m\cdot \exp(-\frac{t}{RC})$$

In Equation (6), m is a constant when the electric field intensity and the motor speed are stable, the current output by the sensor is a square wave, then the voltage of the capacitor in the pre-integral transformation circuit is a stable alternating voltage. Moreover, the characteristics of the output voltage are determined by the parameters of the electric field sensor and the parameters of the pre-integral transformation circuit.

The voltage signal is obtained from the current signal through the pre-integral transformation circuit. When the current signal is a square wave, the voltage signal is a periodic triangular wave. At the end of the *n*-th cycle, set $U_{2}(t\;=\;2T)\;=\;U_{n}$, under steady-state conditions, (The intermediate derivation process is shown in Appendix A). $U_{n}$ is the voltage when t = 0 as shown in Equation (7)

(7) $$U_{n}\;=\;U_{n\;-\;1}=\frac{IR[1\;-\;2\exp(-\frac{T}{RC})+\exp(-\frac{2T}{RC})]}{1\;-\;\exp(-\frac{2T}{RC})}\;=\;IRK$$

In Equation (7), K is a dimensionless constant, as shown in Equation (8)

(8) $$K=\frac{1-2\exp(-\frac{T}{RC})+\exp(-\frac{2T}{RC})}{1-\exp(-\frac{2T}{RC})}$$

Substitute Equation (4) into Equation (8), and the amplitude of the equivalent output voltage of the electric field sensor $U_{n}$ is obtained as shown in Equation (9).

(9) $$U_{n}=4\pi \varepsilon_{0}f_{0}(r_{2}^{2}-r_{1}^{2})RKE$$

According to Equation (9), when $f_{0}$, R, C are determined, the voltage amplitude $U_{n}$ is directly proportional to the electric field intensity E. According to Equations (A2) and (A5) in Appendix A, the output voltage signal within a single cycle is as shown in Equation (10)

(10) $$\left\{\begin{array}{c} U_{1}(t)=-IR[(K+1)\exp(-\frac{t}{RC})-1],0<t<T\\ U_{2}(t)=IR\{[(K+1)-2\exp(\frac{T}{RC})]\exp(-\frac{t}{RC})+1\},T<t<2T \end{array}\right.$$

In Equation (10), $U_{1}(t)$ is the voltage when the induction electrode changes from complete bucking to complete exposure, and $U_{2}(t)$ is the voltage when the induction electrode changes from complete exposure to complete bucking. Equation (10) is the analytical model of the pre-integral transformation circuit.

#### 3.1.2. Parameter Optimization of the Pre-Integral Transformation Circuit

**Figure 8:**
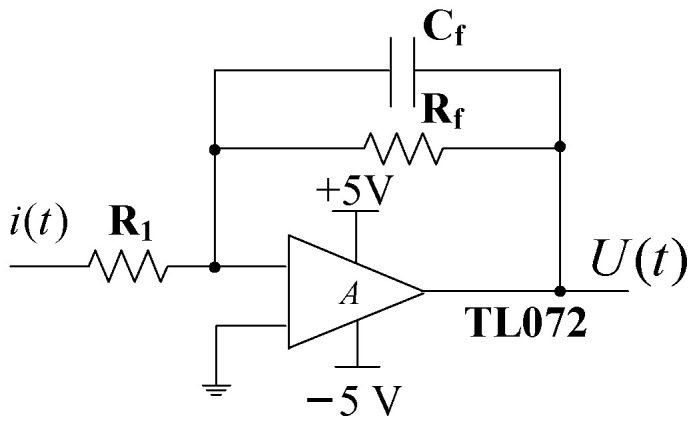
Pre-integral transformation circuit.

The pre-integral transformation circuit adopts a negative feedback current amplification circuit, as shown in Figure 8. $C_{f}$ is a feedback capacitor, and $R_{f}$ provides the − charge discharge path for $C_{f}$. The function of $R_{1}$ is to limit the high-frequency response. The operational amplifier (OPA) adopts the TL072 type amplifier.

**Figure 9:**
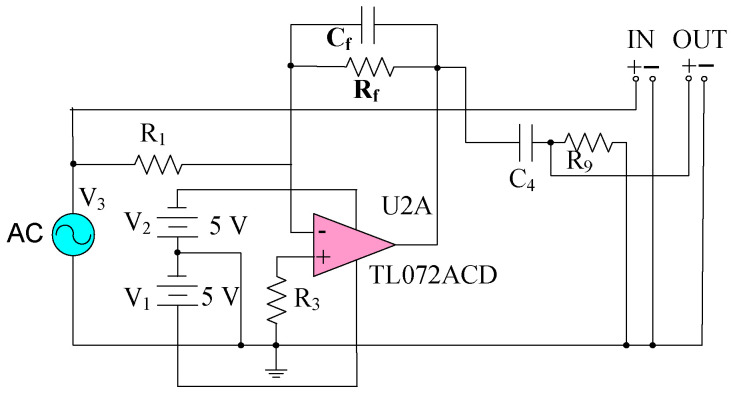
Frequency response simulation circuit.

In order to improve the sensitivity, the frequency response of the circuit is analyzed. When the center frequency of the circuit’s pass band matches the output signal frequency of the sensor’s sensing electrode, the sensitivity of the measurement system is the highest and its anti-interference ability is the strongest. At this time, the optimal $R_{f}$ and $C_{f}$ are obtained. The frequency response simulation circuit is shown in Figure 9.

The passband of the pre-integral transformation circuit is shown in Equation (11):

(11) $$B=f_{H}-f_{L}$$

In Equation (11), B is the bandwidth of the passband, $f_{H}$ is the upper limit frequency, and $f_{L}$ is the lower limit frequency. In order to maximize the system sensitivity, the center frequency $f_{c}$ of the amplitude-frequency response should be adjusted to be the same as the frequency of the sensor output signal. In this device, the rotated frequency of the motor shaft $f_{0}$ is 60 Hz. Since the *Z*-axis adopts four fan-shaped induction electrodes, the main frequency of the output signal generated by the induction electrode (considering the harmonic effect) is four times $f_{0}$, that is, 240 Hz, Since the circuit of this design mainly measures the electric field intensity in the *Z*-axis direction, the center frequency, upper limit frequency and lower limit frequency of the passband are set as $f_{c}$, $f_{H}$ and $f_{L}$ separately, and the expression of component parameters in the circuit is shown in Figure 9 as in (12), and the center frequency $f_{c}$ should be around 240 Hz.

(12) $$\left\{\begin{array}{c} f_{H}=\frac{1}{2\pi C_{f}R_{f}}\\ f_{L}=\frac{1}{2\pi C_{4}R_{3}}\\ f_{c}=\frac{1}{2}(f_{H}+f_{L}) \end{array}\right.$$

**Figure 10:**
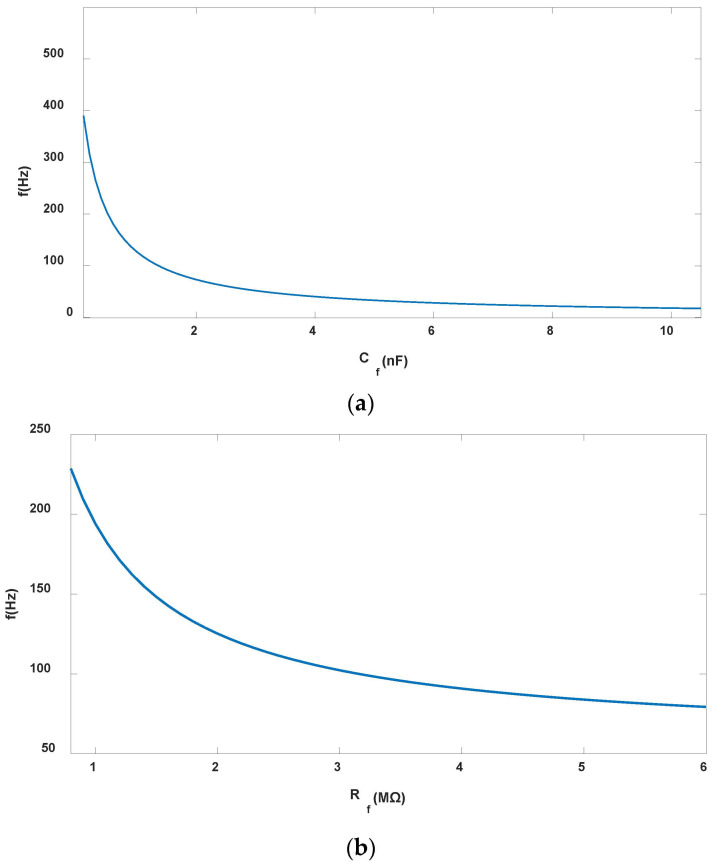
Center frequency: (**a**) Model 1 $R_{f}=1\;M\Omega$; (**b**) Model 2 $C_{f}=0.5\;\mathrm{nF}$.

According to Equation (12), $f_{H}$ and $R_{f}$, $f_{H}$ and $C_{f}$ are inversely proportional. When $R_{f}$ and $C_{f}$ are decreased, $f_{H}$ is increased, when $C_{4}$ and $R_{3}$ remains unchanged, the center frequency $f_{c}$ of the circuit extends towards the high-frequency characteristic and the bandwidth increases. On the contrary, when the $R_{f}$ and $C_{f}$ are increased, $f_{H}$ decreases, the center frequency of the circuit drops and the bandwidth narrows. When $C_{4}$ and $R_{3}$ are constant, $f_{L}$ remains unchanged, when only the values of $R_{f}$ and $C_{f}$ are changed. Two models were selected to study the influence of $R_{f}$ and $C_{f}$ on the circuit response frequency, respectively. In Model 1, $R_{f}=1\;M\Omega$, the influence of $C_{f}$ on *B* and $f_{c}$ is shown in Figure 10a; In Model 2, $C_{f}=\;0.5\;\mathrm{nF}$, the influence of $R_{f}$ on *B* and $f_{c}$ is shown in Figure 10b.

As shown in Figure 10a, when $R_{f}$ remains unchanged, the lager $C_{f}$ is, the lower $f_{c}$ is, and the narrower *B* is. When $C_{f}=0.5\;\mathrm{nF}$, $f_{c}=186\;\mathrm{Hz}$, the requirement that the central frequency $f_{c}$ matches the output frequency of the signals generated by the axial and radial induction electrodes of the sensor is met, and the sensitivity of the measurement system is the highest at this time. Therefore, $C_{f}=0.5\;\mathrm{nF}$ is taken as the quantitative parameter value of Model 2. As Figure 10b shows, when $C_{f}=0.5\;\mathrm{nF}$, the larger $R_{f}$ is, and the narrower *B* is. Therefore, with the $R_{f}$ and $C_{f}$ reduced, $f_{c}$ towards the high frequency, which can improve the sensitivity of the measurement system, the noise generated by the circuit will increase. In this paper, $C_{f}=0.5\;\mathrm{nF}$, $R_{f}=1\;M\Omega$, which can make the sensitivity of the measurement system the highest. At this time, the passband parameters are $f_{c}=186\;\mathrm{Hz}$, $f_{H}=235\;\mathrm{Hz}$, and $f_{L}=137\;\mathrm{Hz}$.

Due to the amplitude $I=4\pi \varepsilon_{0}Ef_{0}(r_{2}^{2}-r_{1}^{2})$ of the induced current generated by the sensor, and $R_{1}$, $R_{f}$, $C_{f}$ of TL072 are the feedback resistance and compensation capacitor between the output and input of the pre-integral transformation circuit. In the steady state, due to the large open-loop gain A of TL072, the equivalent output resistance of the sensor is $R=R_{f}/(1+A)$, and the equivalent output capacitance is $C=(1+A)\cdot C_{f}$. Since A>>1, the output voltage of the pre-integral transformation circuit is shown as Equation (13).

(13) $$U=-4\pi \varepsilon_{0}f_{0}(r_{2}^{2}-r_{1}^{2})R_{f}KE$$

In Equation (13), $r_{1}=7\;\mathrm{mm}$, $r_{2}=16\;\mathrm{mm}$, $f_{0}=60\;\mathrm{Hz}$, $R_{f}=1\;M\Omega$, $T=1/(8\times f_{0})$, E is the measured electric field intensity. K=0.964, which is obtained by Equation (8). The sensor parameters designed in this paper are adopted, $C_{f}=0.5\;\mathrm{nF}$. The output voltage U(t) of the pre-integral transformation circuit within a cycle of 2*T*:

(14) $$U(t)=\left\{\begin{array}{c} IR_{f}[1-(K+1)\exp(-\frac{t}{R_{f}C_{f}})],0<t<T\\ IR_{f}\{[-(K+1)+2\exp(\frac{T}{R_{f}C_{f}})]\exp(-\frac{t}{R_{f}C_{f}})-1\},T<t<2T \end{array}\right.$$

In Equation (14), $C_{f}=0.5\;\mathrm{nF}$, substituting K=0.964 into Equation (14). The amplitude of the induced current output by the sensor I is shown in Equation (15):

(15) $$I=(1.43\;\times \;10^{-12}\left(m/\Omega )\right)\cdot E$$

For the gain of circuit, it mainly depends on the capacitive reactance $X_{\mathrm{Cf}}$ of $C_{f}$ as shown in Equation (15), and the impedance of the feedback resistor $R_{f}$. If $X_{\mathrm{Cf}}<R_{f}$, $C_{f}$ has a great influence on the gain, and if $X_{\mathrm{Cf}}>R_{f}$, $R_{f}$ has a great influence on the gain.

(16) $$X_{Cf}=\frac{1}{2\pi f_{0}C_{f}}=5.134\;M\Omega >R_{f}=1\;M\Omega .$$

Therefore, $R_{f}$ has a significant influence on the gain of the pre-integral transformation circuit, and the gain is $1\;\times \;10^{6}\;V/A$. Then the analytical model of the sensor’s sensing electrode and the pre-integral transformation circuit is as shown in Equation (17):

(17) $$U=(1.43\;\times \;10^{-6}\;m)\cdot E.$$

#### 3.1.3. Noise Analysis of the Pre-Integral Transformation Circuit

**Figure 11:**
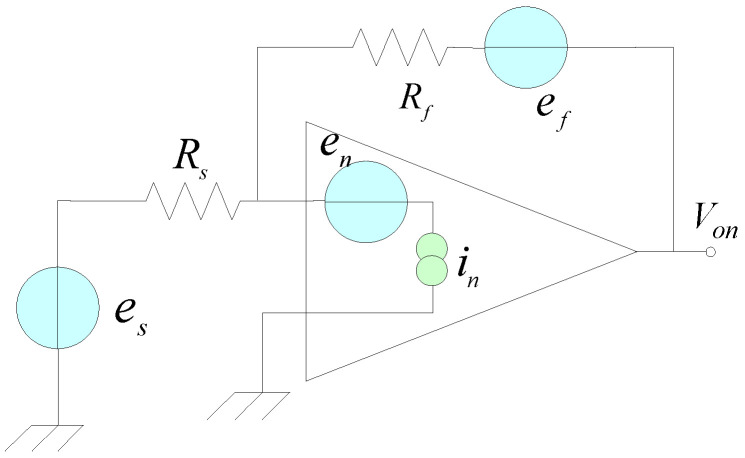
Noise model of the pre-integral transformation circuit.

The noise coefficient of the pre-integral transformation circuit will affect the effect of signal processing. Therefore, it is necessary to analyze the noise model of the circuit; the noise model is shown in Figure 11:

In Figure 11, the noise sources include the equivalent input noise voltage $e_{n}$, equivalent input noise current $i_{n}$, the thermal noise $e_{f}$ of resistance $R_{f}$, the thermal noise $e_{s}$ generated by the signal source internal resistance $R_{s}$, and Johnson noise $V_{n}$ are shown in Equation (18).

(18) $$V_{n}=\sqrt{4kT’RB}$$

In Equation (18), k is Boltzmann constant, $k=1.38\;\times \;10^{-23}\;J/K$, T’ is the absolute temperature. In this paper, T’=300 K. Ignoring the mutual influence of the noise generated by electronic components, calculate the results of each noise acting independently, and the total noise output voltage is calculated based on the superposition principle. The total noise is the sum of each noise source, as shown in Equation (19):

(19) $$V_{\mathrm{on}}=\sqrt{\int_{f_{L}}^{f_{H}}[(i_{f}^{2}+i_{n}^{2}){\left|\frac{R_{f}}{1+j\omega R_{f}C_{f}}\right|}^{2}+e_{n}^{2}]df}.$$

In Equation (19), $i_{f}=\sqrt{4kT/R_{f}}A/\sqrt{\mathrm{Hz}}$, which is the noise current spectral density of the feedback resistor, $R_{f}=1\;M\Omega \;=\;R$, $i_{f}=1.29\;\times \;10^{-13}A/\sqrt{\mathrm{Hz}}$, because $f_{L}=235\;\mathrm{Hz}$ $f_{H}=137\;\mathrm{Hz}$. In the case of the maximum noise, $e_{n}=18\;\mathrm{nV}/(\mathrm{Hz}{)}^{\frac{1}{2}}$. Substitute the above parameters into Equation (19), where the amplitude of the noise is $V_{\mathrm{on}}\approx 2\;\mu V$. The electric field intensity on a sunny day is much less than that on a thunderstorm day. If the electric field intensity on a sunny day is 100 V/m, the output voltage amplitude of the pre-integral transformation circuit is $1.43\;\times \;10^{-4}\;V$, which is more than 70 times the amplitude of the noise signal and much greater than the noise amplitude. The circuit is less disturbed by thermal noise, and the noise has little influence on the voltage signal output by the sensor.

### 3.2. Parameters for Differential Amplification Circuit

When measuring the atmospheric electric field on a sunny day, the electric field intensity E is within the range $\left[100\;V/m,\;150\;V/m\right]$. By the sensor and the pre-integral transformation circuit, the amplitude of the obtained voltage signal is between $1.4\;\times \;10^{-4}\;\mathrm{mV}∼3\;\times \;10^{-4}\;\mathrm{mV}$, the signal still cannot undergo A/D conversion, so a differential amplification circuit is needed to further amplify it. The differential amplification circuit adopts AD620, with an external resistor $R_{4}$ added. The gain is set within the range of 1 to 10,000 times, as shown in Equation (20):

(20) $$R_{4}=\frac{49.4\;k\Omega }{G-1}.$$

**Figure 12:**
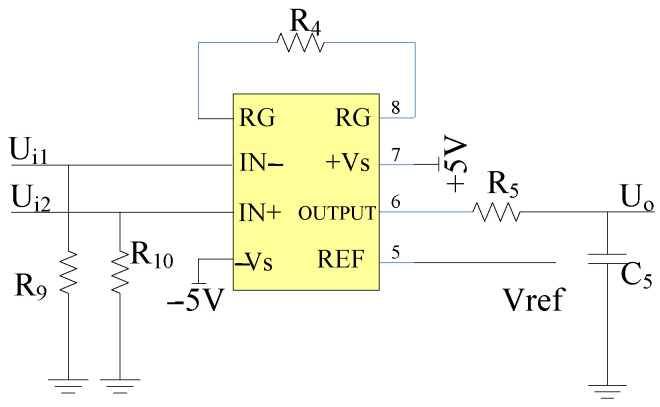
Differential amplification circuit.

In Equation (20), G represents the gain of the differential amplification circuit; due to the high input impedance of the AD620, the input voltage is highly sensitive to the bias current. At the two differential input ports, unloading resistance $R_{9}$ and $R_{10}$ are added, respectively. The principle of the differential amplification circuit is shown in Figure 12.

In Figure 12, $R_{9}$ is the same resistor as $R_{9}$ in Figure 9, and both $R_{9}$ and $R_{10}$ are part of the high-pass filter.

Since the A/D converter of the measurement system is a 12-bit A/D converter in ARM, the voltage range of A/D conversion is [0,3.3 V]. The output amplitude of AD620 is limited by the power supply voltage. According to the AD620 manual, the maximum output voltage should be $U_{s}-1.1$, where $U_{s}$ is 5 V. Therefore, $U_{s}-1.1=3.9\;V$, which is greater than 3.3 V and meets the requirements. The signal output by the sensor is amplified by the pre-integral transformation circuit and the differential amplification circuit, which is within this measurement range as $U_{\max}=3.3\;V$. The theoretical minimum resolution of the A/D conversion Res is shown in Equation (21):

(21) $$\mathrm{Res}=\frac{U_{\max}}{2^{12}}=0.806\;\mathrm{mV}.$$

Since the current output by the induction electrode is usually at the pA level, it needs to be amplified to the mV level by the amplifier circuit before the conditions for A/D conversion can be met. As Equation (17) shows, the amplitude of the output voltage signal generated by the pre-integral transformation circuit is $(1.43\times 10^{-6}\;m)\;\cdot E$, when the measurement system’s range is 20 kV/m, the output signal amplitude of the pre-integral transformation circuit should theoretically be 28.6 mV. As the maximum output voltage amplitude of 1.6 V, the maximum amplification factor of 1.6 V/28.6 mV = 55.94 times is obtained. Considering that the signal is interfered by harmonics and DC components, the amplitude of the signal after differential amplification according to the maximum amplification factor will be greater than the range. Therefore, the selection of the amplification factor needs to be lower. The differential amplification factor is selected as 30. From Equation (20), it can be known that the external resistance $R_{4}=1.7\;k\Omega$.

When the differential amplification factor is selected as 30, the measurement range of the sensor is 20 kV/m. When an electric field intensity of 20 kV/m is applied, the theoretical amplitude of the output voltage signal of the sensor (the amplitude of the 4th harmonic) is 0.858 V. The resolution of the sensor for measuring the electric field intensity is shown in Equation (22), meeting the measurement accuracy requirements.

(22) $$\mathrm{Res}\prime \;=\;\frac{\mathrm{Res}}{30\;\times \;1.43\;\times \;10^{-6}}\;=\;18.7\;V/m$$

### 3.3. Biasing Circuit

**Figure 13:**
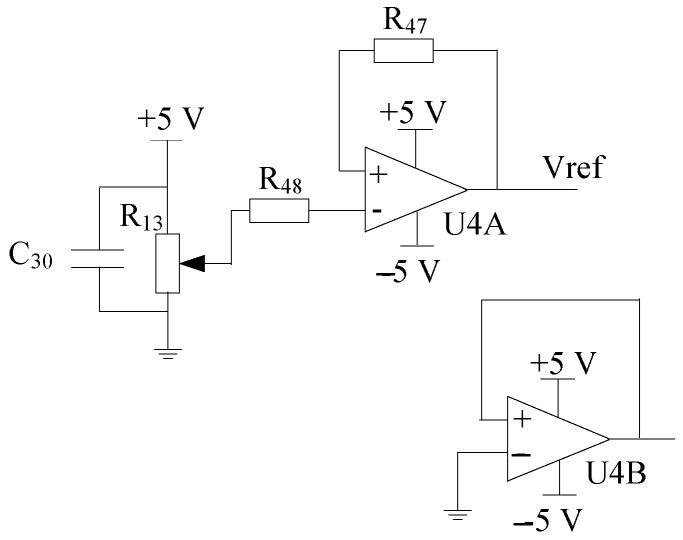
Biasing circuit.

The effective voltage for A/D conversion is 0–3.3 V, and the amplitude of the amplified signal needs to be within the full scale range. The function of the bias circuit is to shift the output voltage of the differential amplifier circuit upward along the *Y*-axis. Therefore, the bias circuit sets the reference voltage terminal REF of AD620 to 1.6 V by adjusting $R_{13}$, and performs FFT harmonic decomposition and effective A/D conversion. The principle is shown in Figure 13.

## 4. Harmonic Amplitude Detection Method Based on the Improved FFT-BP

### 4.1. Improved FFT-BP

**Figure 14:**
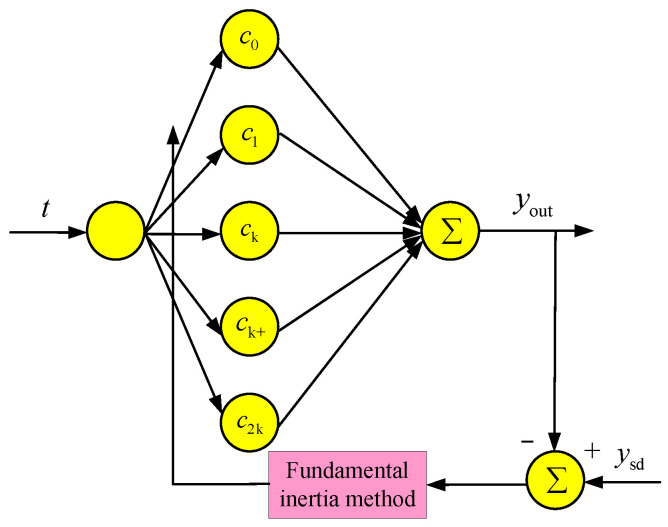
FFT-BP neural network.

Since the voltage signal output by the sensor contains DC components, harmonics, and noise interference, a harmonic amplitude detection method based on the improved FFT-BP is proposed in this paper. The harmonic parameter information obtained by FFT preprocessing is used to limit the search area of the BP network parameter iteration. Meanwhile, the learning rate, momentum factor, and excitation function jointly regulate the BP network. The improved BP nerve is shown in Figure 14.

$t_{i}(i\;=\;1,\;2,\;3\ldots ,\;N)$ is the input of the BP neural network, N is the total number of training samples, and the excitation function of the neuron is shown in Equation (23):

(23) $$c(t)\;=\;[\sin(k_{1}\omega t\;+\;\varphi_{1}),\;\sin(k_{2}\omega t\;+\;\varphi_{2}),\cdots ,\sin(k_{n}\omega t+\varphi_{n})].$$

In Equation (23), *n* is the highest-order harmonic, $k_{l}(l\;=\;1,2,\ldots ,N)$ is the order of the harmonic, and $\varphi_{l}(l\;=\;1,\;2,\;\ldots ,\;N)$ is the phase of the harmonic, $k_{l}(l\;=\;1,\;2,\;\ldots ,\;N)$ and $\varphi_{l}(l\;=\;1,\;2,\;\ldots ,N)$ are variable parameters. The output of the BP neural network $y_{\mathrm{out}}(t_{i})$ is shown in Equation (24).

(24) $$y_{\mathrm{out}}(t_{i})\;=\;\sum_{l=i}^{n}A_{l}c_{l}(t_{i})\;=\;\sum_{l=i}^{n}A_{l}\sin(k_{l}\omega t_{i}\;+\;\varphi_{l})$$

In Equation (24), i=1, 2,⋯, N, error e(i) is the difference between input $y_{\mathrm{sd}}$ and output $y_{\mathrm{out}}$ as shown in Equation (25).

(25) $$e(i)\;=\;y_{\mathrm{sd}}\;-\;y_{\mathrm{out}}$$

The training index V of the BP neural network is as shown in Equation (26):

(26) $$V\;=\;\sum_{i=1}^{n}{\left[e(i)\right]}^{2}.$$

The basic inertia algorithm is taken as the excitation function and weight learning algorithm in the BP network, as shown in Equation (27):

(27) $$\left\{\begin{array}{c} ∆A_{l}(i)\;=\;-\eta_{A}\frac{\partial V(i)}{\partial A_{l}}\;+\;\alpha_{A}∆A_{l}(i\;-\;1)\\ ∆k_{l}(i)\;=\;-\eta_{k}\frac{\partial V(i)}{\partial k_{l}}\;+\;\alpha_{k}∆k_{l}(i\;-\;1)\\ ∆\varphi_{l}(i)\;=\;-\eta_{\varphi }\frac{\partial V(i)}{\partial \varphi_{l}}\;+\;\alpha_{\varphi }∆\varphi_{l}(i\;-\;1) \end{array}\right..$$

In Equation (27), η and α are the learning rate and momentum factor, respectively, which determine the convergence speed of the BP network. If the value is too little, the number of iterations will be too many and the calculation speed will be slow. If the value is too large, the iteration will fall into a local minimum, which is specifically manifested as oscillating near the convergence point and network failure. During the training process, based on the partial derivatives of the error and the values obtained in the previous iteration, η and α are adjusted, and the resulting weight vector is as shown in Equation (28):

(28) $$\left\{\begin{array}{c} A\;=\;\left[A_{1},\;A_{2},\ldots ,\;A_{n}\right]\\ k\;=\;\left[k_{1},\;k_{2},\ldots ,\;k_{n}\right]\\ \varphi \;=\;\left[\varphi_{1},\;\varphi_{2},\ldots ,\;\varphi_{n}\right] \end{array}\right..$$

In Equation (28), $A_{l}$, $k_{l}$ and $\varphi_{l}$ are the amplitudes, frequencies, and phases of the l-th harmonic, respectively. Based on the traditional FFT-BP algorithm, an improved FFT-BP algorithm is proposed, the concept of threshold interval was introduced, the frequency, amplitude, and phase are, respectively, limited within [k − ∆k, k + ∆k], [A − ∆A, A + ∆A], and [φ − ∆φ, φ + ∆φ]. The interval range is adjusted according to the accuracy of FFT preprocessing in the actual application situation. The adjustment rules are shown in Equation (29).

(29) $$\left\{\begin{array}{c} ∆A\;\leq \;\delta A\\ ∆k\;\leq \;\delta k\\ ∆\varphi \;\leq \;\delta \varphi \end{array}\right.$$

In Equation (29), δA, δk, and δφ are the detection accuracies of FFT for amplitude, frequency, and phase, respectively, δA, δk, and δφ can be set as needed. After optimization, the number of iterations can be reduced, the iteration operation time can be shortened, and the real-time performance of the algorithm can be improved. The ideal signal containing the 9th harmonic with additional Gaussian white noise (20 dB) was decomposed, respectively, by using the traditional FFT-BP method and the improved FFT-BP method. The signals containing the first to the ninth harmonics are shown in Equation (30):

(30) $$\begin{array}{l} x\;=\;100\sin(120\pi t\;+\;\frac{\pi }{3})\;+\;90\sin(240\pi t\;-\;\frac{\pi }{3})\;+\;80\sin(360\pi t\;+\;\frac{\pi }{3})\;+\;\\ 70\sin(480\pi t\;-\;\frac{\pi }{3})\;+\;60\sin(600\pi t+\frac{\pi }{3})+50\sin(720\pi t\;-\;\frac{\pi }{3})\;+\;\\ 40\sin(840\pi t\;+\;\frac{\pi }{3})\;+\;30\sin(960\pi t\;-\;\frac{\pi }{3})\;+\;20\sin(1080\pi t\;+\;\frac{\pi }{3}) \end{array}.$$

In the FFT-BP algorithm, the number of neurons is 5, l = 9, the fundamental frequency is 60 Hz, the initial value of $\eta_{k}$ and $\eta_{A}$ in the BP neural network are 0.01, and the initial value of $\eta_{\varphi }$ is 0.02, $\alpha_{A}\;=\;\alpha_{k}\;=\;\alpha_{\varphi }\;=\;0.5$. The improved algorithm is adjusted according to the rule of Equation (29). Since the device studied in this paper mainly measures the amplitude and frequency of the signal, the accuracy requirements for amplitude and frequency detection are relatively high. δA, δk, and δφ are set as 0.001, 0.005, and 0.01. The comparisons of signal amplitudes and errors obtained by the two algorithms are shown in Table 1.

**Figure 15:**
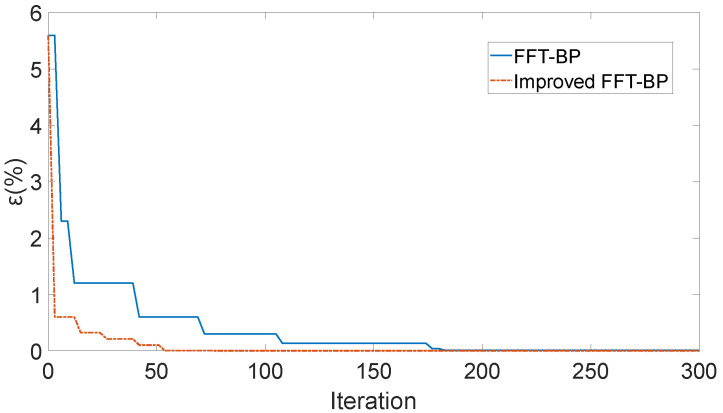
The relationship between the number of iterations and the amplitude error.

As shown in Table 1, with the harmonic order increases, the errors of the two algorithms increased. Compared with the traditional FFT-BP method, the error change of the improved FFT-BP algorithm was relatively slow, and the error of the improved algorithm was slightly less than that before the improvement. The relationship between the amplitude error and the number of iterations is shown in Figure 15.

As shown in Figure 15, the traditional FFT-BP algorithm reaches the minimum and remains unchanged after iteration, while the improved FFT-BP algorithm only needs to be run with 55 iterations, with less operation time and fewer iterations, improving the real-time performance of the algorithm. Further analysis of the relationship between the error and the harmonic order. By changing the amplitude of the ideal signal, the amplitudes of all harmonic signals are 100. The signal containing noise is shown in Equation (31), and the noise is still 20 dB white noise. The comparison of the signal amplitudes and errors obtained by the two algorithms is shown in Table 2:

(31) $$\begin{array}{l} x\;=\;100[\sin(120\pi t\;+\;\frac{\pi }{3})\;+\;\sin(240\pi t\;-\;\frac{\pi }{3})\;+\;\sin(360\pi t\;+\;\frac{\pi }{3})\;+\;\sin(480\pi t\;-\;\frac{\pi }{3})\;+\;\\ \sin(600\pi t\;+\;\frac{\pi }{3})\;+\;\sin(720\pi t\;-\;\frac{\pi }{3})\;+\;\sin(840\pi t\;+\;\frac{\pi }{3})\;+\;\sin(960\pi t\;-\;\frac{\pi }{3})\;+\;\sin(1080\pi t\;+\;\frac{\pi }{3})] \end{array}$$

As Table 2 shows, when the traditional FFT-BP algorithm was used, the relative error is between 0.3% and 1.6%, and the errors of the high-frequency components (such as 480 Hz and 540 Hz) are slightly bigger. However, when the improved FFT-BP algorithm was used to detect the amplitude of each harmonic, the error was controlled within 0.06–0.12%, and the accuracy is higher than that of the traditional FFT-BP algorithm. The error increases slowly with the increase of the harmonic order. The improved FFT-BP algorithm has higher detection accuracy for the amplitudes of each component of the harmonic.

**Figure 16:**
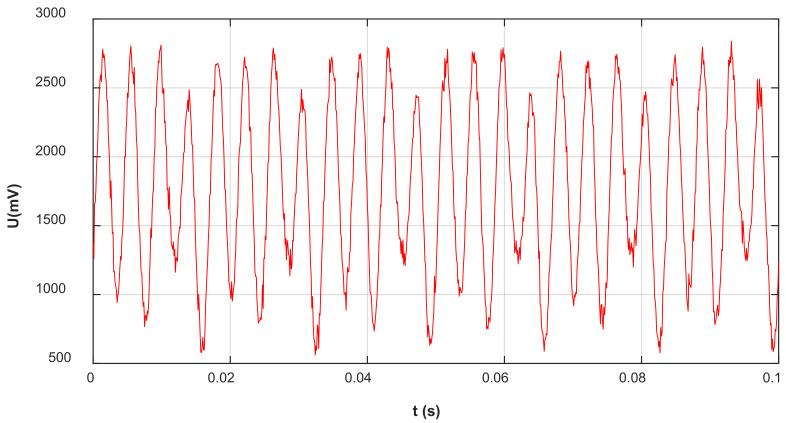
*Z*-axis induction electrode output signal.

When the sensor is placed in a uniform electric field parallel to the *Z*-axis (E = 20 kV/m), the output signal generated from the *Z*-axis induction electrode is shown in Figure 16. The signal amplitude is within the range [−1 V, +1 V]. Under the action of the bias circuit, the signal increased by 1.6 V. The new signal amplitude range is [0.6 V, +2.6 V], within the range of [0, +3.3 V], can meet the requirements of A/D conversion. The above-mentioned trained model is adopted to optimize the sensor output signal after FFT decomposition and filtering.

**Figure 17:**
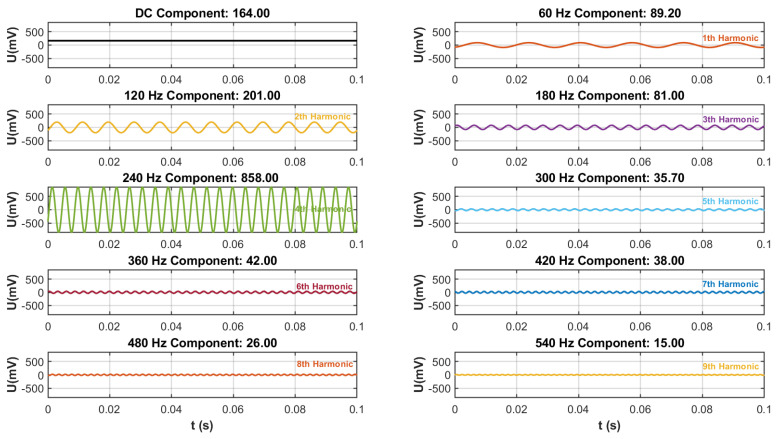
Signal component.

The rotated frequency of the motor in the sensor is 60 Hz, so the fundamental frequency of the output signal was also 60 Hz. Due to the *Z*-axis induction electrode adopting a four-sector structure, theoretically, the frequency of its output signal was four times the motor frequency, which is the fourth harmonic frequency of 240 Hz. The improved FFT-BP method was adopted, with each parameter remaining unchanged; the signal generated by the Z-direction induction electrode of the sensor was decomposed. Among them, the DC component needs to be subtracted by 1.6 V (generated by the bias circuit). The signals at each frequency are shown in Figure 17.

**Figure 18:**
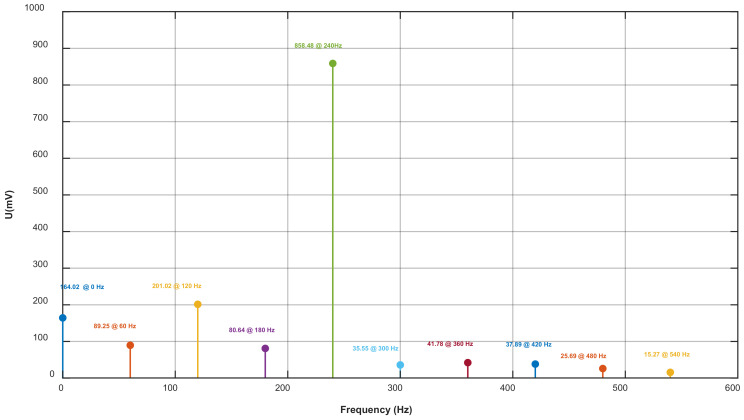
Relationship between signal frequency and amplitude obtained by FFT-BP.

As Figure 17 shows, after the harmonics were decomposed by the improved FFT-BP method, the signal was divided into 10 groups of signals of different frequencies. Among them, the amplitude of the 4th harmonic (240 Hz) was the highest as 858.48 mV, which was the main output signal of the Z induction electrode, the fundamental wave signal was 89.25 mV and the DC component is 164.02 mV. The relationship between the amplitudes and frequencies of each component signal is shown in Figure 18.

### 4.2. Measurement Experiment

**Figure 19:**
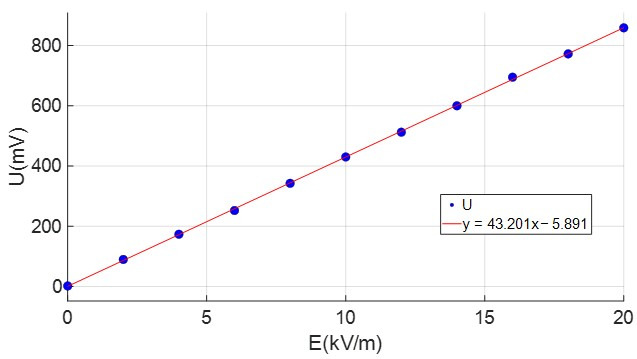
The relationship between *U* and *E.*

The optimized sensor was calibrated. Taking the *Z*-axis induction electrode as the research object, the sensor was placed in a uniform electric field with adjustable intensity. The direction of the uniform electric field was parallel to the *Z*-axis. The relationship between the output voltage amplitude of the electrode in the *Z*-axis direction and the electric field intensity is shown in Figure 19.

As Figure 19 shows, the value of the electric field intensity measured by the sensor was basically consistent with the actual loaded electric field intensity by the parallel plate capacitor. The determination coefficient of the measured data $R^{2}\;=\;0.997$ was approximately 1, which had good linearity. The resolution for measuring the atmospheric electric field strength can reach 18.7 V/m, and the sensitivity was 42.9 mv/(kV/m).

## 5. Design of Power Supply System for Electric Field Sensor

### 5.1. The Design of the Power Module of the System

In order to achieve accurate detection of atmospheric electric field intensity, on the basis of completing the design of the electric field sensor, the design of the system power supply is completed. According to the power supply voltages required by different modules, there are three types of power supplies used in the system: the power supply for the signal amplification circuit (analog circuit), the power supply for the digital circuit, and the power supply for the brushless DC motor that drives the electrodes to rotate.

(1) A power supply for amplifying the analog signal output by the sensor

This section adopts 8 V DC input voltages. Since the operational amplifier chips TL072 and AD620 in the signal amplification circuit section were powered by dual power supplies. Four 3.7 V, 600 mAh lithium batteries were connected in series, and a ground wire was led out from the middle to form the 3.7 V and power supply voltage. The voltage of this type of lithium battery when fully charged was 4.4 V. The maximum working voltage of TL072 was ±18 V, and the working voltage range of AD620 was ±2.3 V to ±18 V. Therefore, the 8 V power module can meet the requirements.

(2) Power supply for digital circuits

The digital circuit part adopted two DC voltages of 3.3 V and 1.6 V; the 3.3 V DC voltage is the working voltage of the main controller. The main controller of this system selects the STM32F103RC chip of the STM32F103 series of ARM single-chip microcomputer, which had a faster operating speed, lower power consumption, and more abundant port resources. It can not only achieve precise and rapid measurement of electric field intensity, and its abundant port resources can add new functions to the measurement system, such as adding GPS modules, three-dimensional electronic compass modules, wireless transceiver modules, which can provide necessary conditions for the system to adopt the three-dimensional electric field intensity of the air atmosphere in the unmanned aerial vehicle carrier mode.

For the voltage required for A/D conversion of the single-chip microcomputer, a 1.6 V DC voltage was selected in this paper. In this paper, the TL072 integrated operational amplifier chip and potentiometer were used to implement the 1.6 V reference voltage module. The effective voltage for AD conversion was 0–3.3 V. Adjusting the potentiometer to the position where the DC output voltage is 1.6 V can meet the conversion conditions for full-scale AD conversion.

(3) Power supply for brushless DC motors

In order to achieve the measurement of the atmospheric electric field intensity in the air under the airborne mode of the unmanned aerial vehicle in the subsequent work, this device adopted 12 V DC voltage as the power supply voltage for the DC motor. It used a large-capacity rechargeable lithium battery of 12 V/1800 mAh. After charging, the motor can work continuously for more than 5 h, and the input voltage of the battery was 12.6 V. The output voltage was 10.8–12.6 V, which could realize the measurement of the atmospheric electric field intensity in the air in the airborne mode.

### 5.2. Comparison of the Main Performance Parameters of Sensors

The main performance parameters of the sensor designed in this paper were compared with those of the sensor designed in references [3,4,10]. The comparison of relevant parameters is shown in Table 3.

As Table 3 shows, the sensor and signal processing system designed in this paper had an induction electrode diameter of 3.2 cm. Compared with the previous several sensors, it was more lightweight. The measurement range was ±20 kV/m, which was more suitable for the measurement of atmospheric electric field intensity. The resolution was 18.7 V/m, which is higher than the 30 V/m proposed in Reference [4]. The sensitivity of the sensor studied in this paper is 42.9 mv/(kV/m), which was higher than that in Reference [3] which was 1 mV/(V/m), 48.75 mv/(kV/m) in References [4,10], where the sensitivity was 45 mv/(kV/m), and the sensitivity had been improved to a certain extent. The power supply system adopts multiple different voltages for the power supply, making the power supply more stable.

### 5.3. Atmospheric Electric Field Intensity Measurement Experiment

**Figure 20:**
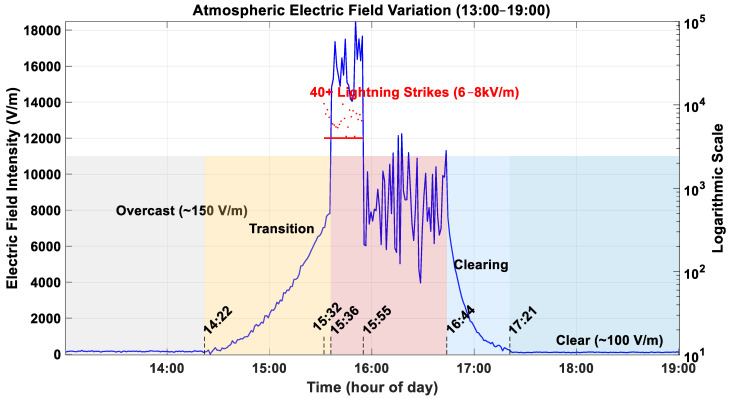
Measured data on 27 March 2025.

The atmospheric electric field intensity was measured by using the optimized electric field sensor. The measured data at Huzhou City, Zhejiang Province, China (30.87315 N, 120.12456 E) from 1 a.m. to 7 a.m. on 27 March 2025 was shown in Figure 20, the weather will be cloudy turning to moderate rain, with an ambient temperature ranging from 16 °C to 18 °C and a relative humidity of 60%, the sensor was installed in an open area. As the results show, the atmospheric electric field fluctuates around 150 V/m on cloudy days and around 100 V/m on sunny days. The electric field intensity changes significantly from 2:22 to 5:21, especially from 3:32 to 3:55, with more than 40 lightning strikes occurring continuously, which cause the amplitude of the electric field intensity to fluctuate sharply between 12 and 18 kV/m. The date is consistent with the yellow lightning warning issued by the Zhejiang Meteorological Bureau on the same day, and the occurrence of lightning activities was observed on the spot.

## 6. Conclusions

Aiming at the issues that the field mill type electric field sensor lacks an accurate and complete mathematical model, and its signal is weak and contains a large amount of harmonic noise, the parameters of the axial electrode structure, signal processing methods and signal processing circuits of the field grinding electric field sensor are studied and optimized in this paper.

(1) By analyzing the measurement principle of the field grinding electric field sensor, a mathematical model of the sensor’s induction electrode was established. Due to the edge effect of the electric field, the finite element analysis method was adopted, and the effect law that the thickness of the sensor bucking electrode h and the distance between the bucking electrode and the induction electrode d on the change of its surface charge were analyzed. On this basis, the influences of d and h on the sensor sensitivity were analyzed. Considering the sensitivity, the installation and manufacturing cost of the electrode comprehensively, the optimized structural parameters obtained are: d = 2 mm, h = 1 mm, $r_{1}\;=\;6\;\mathrm{mm}$, $r_{2}\;=\;17\;\mathrm{mm}$.
(2) Aiming at the problem that the amplitude of the output signal of the sensor is weak, and effective amplitude measurement cannot be carried out, a weak signal amplification circuit including a pre-integral transformation circuit, a differential amplification circuit, and a biasing circuit was designed and studied. Firstly, the equivalent analytical model of the pre-integral transformation circuit was established, the effect of the feedback resistor $R_{f}$ and the integrating capacitor $C_{f}$ on the passband of the circuit was analyzed, according to the characteristics of the output signal generated by the sensor, the optimized parameters $C_{f}\;=\;0.5\;\mathrm{nF}$ and $R_{f}\;=\;1\;M\Omega$ were obtained, and the passband was controlled at $f_{H}\;=\;235\;\mathrm{Hz}$, $f_{L}\;=\;137\;\mathrm{Hz}$, the center frequency $f_{C}\;=\;186\;\mathrm{Hz}$. The analytical model of pre-integral transformation circuit that $U\;=\;(1.43\;\times \;10^{-6}\;m)\cdot E$ was obtained by the optimized parameters, the sensitivity of the circuit was improved, the measurement resolution of the electric field intensity was 18.7 V/m. A noise model for the pre-integral transformation circuit was studied, under the action of the electric field in sunny conditions, the amplitude of the generated voltage signal was more than 70 times that of the noise signal, and the interference of thermal noise to the measurement system was reduced effectively. Meanwhile, a differential amplification circuit and a bias circuit were designed to meet the A/D conversion requirements.
(3) An improved FFT-BP algorithm was proposed to detect the harmonic amplitude of the sensor output signal. Based on the parameters obtained from the FFT for harmonic preprocessing and setting the parameters of the BP network, the parameter iteration and search range of the BP network are restricted. The learning factor, momentum factor, and activation function are used to jointly adjust the BP network. The simulation results show that the harmonic amplitudes obtained by this method are more accurate than those of the traditional FFT method, and the amplitude error is controlled between 0.06% and 0.12%. Calibration experiments were conducted under a uniform electric field of known intensity, within the range of electric field intensity of 0~20 kV/m, the determinability coefficient of the system is $R^{2}\;=\;0.997$, and the resolution is 18.7 V/m, which can meet the measurement requirements. The actual environment was measured by using the optimized device in this paper. The measurement results show that the measurement system can measure the atmospheric electric field and provide reliable measurement data for lightning monitoring and early warning.
(4) By comparing the sensors studied in references [3,4,10], the sensors and their signal processing circuits studied in this paper are smaller in size and lighter in weight, and are more suitable for the aerial electric field measurement in the airborne mode of unmanned aerial vehicles. The parameters such as the resolution and sensitivity of the device studied in this paper are slightly due to those in references [3,4,10], etc. However, the device designed in this paper has more modules and a more complex power supply system.

## Figures and Tables

**Table 1:** Comparison of signal amplitude and error with different amplitudes.

Order of Harmonics	Theoretical Amplitude	Traditional FFT-BP		Improved FFT-BP	
		Measured Amplitude	Error (%)	Measured Amplitude	Error (%)
1	100	99.87	0.13	100.06	0.060
2	90	89.92	0.09	89.993	0.080
3	80	80.15	0.19	79.9956	0.055
4	70	69.78	0.31	70.042	0.060
5	60	60.33	0.55	59.952	0.081
6	50	49.65	0.70	50.042	0.082
7	40	40.36	0.90	40.035	0.087
8	30	29.71	0.97	30.025	0.086
9	20	20.28	1.40	19.979	0.105

**Table 2:** Comparison of signal amplitude and error with the same amplitude.

The Order of Harmonics	Traditional FFT-BP		Improved FFT-BP	
	Measured Amplitude	Error (%)	Measured Amplitude	Error (%)
1	99.12	0.88	100.08	0.08
2	100.31	0.31	99.96	0.06
3	99.67	0.33	99.94	0.06
4	101.05	1.05	100.07	0.07
5	98.92	1.08	99.93	0.07
6	99.34	0.66	100.09	0.09
7	100.72	0.72	100.11	0.11
8	98.45	1.55	100.11	0.11
9	101.18	1.18	99.88	0.12

**Table 3:** Comparison of the main performance parameters of the sensor.

Parameter	Reference [3]	Reference [4]	Reference [10]	This Work
Number of sector-shaped vanes	2	6	2	4
Sensor dimensions	3 cm	N/A	15.2 cm	3.2 cm
Motor type	N/A	Brushless DC motor	Brushless DC motor	Brushless DC motor
Rotation frequency, f (Hz)	60	N/A	12.75	60
Electric field range	150 V/m	0–80 kV/m	±20 kV/m	±20 kV/m
Resolution	16 bits	30 V/m	16 bit	16 bits 18.7 V/m
Sensitivity	~1 mV/(V/m)	48.75 mV/(kV/m)	45 mV/(kV/m)	42.9 mV/(kV/m)
Power supply	6 V	4 V	3 V analog front-end supply 5 V motor supply	3 V analog front-end supply 1.6 V supply for digital circuit 12 V motor supply

## Data Availability

The data are available from the corresponding author on reasonable request.

## Abbreviations

The following abbreviations are used in this manuscript:

FFT	fast Fourier transform
BP	back propagation neural network
DC	direct current
HHT	Hilbert–Huang transform
Ap-FFT	all-phase fast Fourier transform
OPA	operational amplifier
A/D	analog to digital

## Appendix A

Set the output voltage to be $U_{n-1}$ at the end of the (*n* − 1)-th period, that is, when t=0, and $U_{n}$ at the end of *n − t* that is, t=2T. As $I_{Z}(t)=\left\{\begin{array}{c} \frac{dQ_{Z}(t)}{dt}=-4\pi \varepsilon E_{Z}f_{0}(r_{2}^{2}-r_{1}^{2}),0<t<T\\ \frac{dQ_{Z}(t)}{dt}=4\pi \varepsilon E_{Z}f_{0}(r_{2}^{2}-r_{1}^{2}),T<t<2T \end{array}\right.$, when 0<t<T, I(t)=−I, substituting into $U(t)=I\cdot R-m\cdot \exp(-\frac{t}{RC})$, $U_{1}(t)$ is shown as Equation (A1)

(A1) $$U_{1}(t)=-I\cdot R+m_{1}\cdot \exp(-\frac{t}{RC}),0<t<T$$

When t=0, $m_{1}$ is a constant. Since $U_{1}(t=0)=U_{n-1}=-IR+m_{1}$, then $m_{1}=U_{n-1}+IR$. Therefore, when 0<t<T, $U_{1}(t)$ is shown in Equation (A2):

(A2) $$U_{1}(t)=-IR+(U_{n-1}+IR)\exp(-\frac{t}{RC}),0<t<T$$

Similarly, when T<t<2T, I(t)=+I, $U_{2}(t)$ is shown in Equation (A3):

(A3) $$U_{2}(t)=IR+m_{2}\exp(-\frac{t}{RC}),T<t<2T$$

In Equation (9), $m_{2}$ is a constant. when t=T, based on the consistency of the signal amplitude, $U_{1}(t)=U_{2}(t)$, as shown in Equation (A4):

(A4) $$IR+m_{2}\exp(-\frac{T}{RC})=-IR+(U_{n-1}+IR)\exp(-\frac{T}{RC})$$

In Equation (10), $m_{2}=-2IR\exp(\frac{T}{RC})+U_{n-1}+IR$. Substitute $m_{2}$ into Equation (A5),

(A5) $$U_{2}(t)=IR+[U_{n-1}+IR-2IR\exp(\frac{T}{RC})]\exp(-\frac{t}{RC}),T<t<2T$$

At the end of the n-th cycle, $U_{2}(t=2T)=U_{n}$.
